# Fibrates for the Treatment of Primary Biliary Cholangitis Unresponsive to Ursodeoxycholic Acid: An Exploratory Study

**DOI:** 10.3389/fphar.2021.818089

**Published:** 2022-01-20

**Authors:** Guilherme Grossi Lopes Cançado, Cláudia Alves Couto, Laura Vilar Guedes, Michelle Harriz Braga, Débora Raquel Benedita Terrabuio, Eduardo Luiz Rachid Cançado, Maria Lucia Gomes Ferraz, Cristiane Alves Villela-Nogueira, Mateus Jorge Nardelli, Luciana Costa Faria, Elze Maria Gomes de Oliveira, Vivian Rotman, Daniel Ferraz de Campos Mazo, Valéria Ferreira de Almeida e Borges, Liliana Sampaio Costa Mendes, Liana Codes, Mario Guimarães Pessoa, Izabelle Venturini Signorelli, Cynthia Levy, Paulo Lisboa Bittencourt

**Affiliations:** ^1^ Instituto Alfa de Gastroenterologia, Hospital das Clínicas da Universidade Federal de Minas Gerais, Belo Horizonte, Brazil; ^2^ Hospital da Polícia Militar de Minas Gerais, Belo Horizonte, Brazil; ^3^ Departamento de Gastroenterologia, Faculdade de Medicina da Universidade de São Paulo, São Paulo, Brazil; ^4^ Disciplina de Gastroenterologia, Universidade Federal de São Paulo, São Paulo, Brazil; ^5^ Hospital Universitário Clementino Fraga Filho e Departamento de Clínica Médica da Faculdade de Medicina, Universidade Federal do Rio de Janeiro, Rio de Janeiro, Brazil; ^6^ Centro Universitário Lusíada—UNILUS, Santos, Brazil; ^7^ Divisão de Gastroenterologia (Gastrocentro), Faculdade de Ciências Médicas, Universidade Estadual de Campinas, Campinas, Brazil; ^8^ Instituto de Gastroenterologia, Endoscopia e Proctologia, Uberlândia, Brazil; ^9^ Universidade Federal de Uberlândia, Uberlândia, Brazil; ^10^ Hospital de Base do Distrito Federal, Brasília, Brazil; ^11^ Hospital Universitário Professor Edgard Santos, Universidade Federal da Bahia, Salvador, Brazil; ^12^ Hospital Português, Salvador, Brazil; ^13^ Hospital Universitário Cassiano Antônio Moraes, Universidade Federal do Espírito Santo, Vitória, Brazil; ^14^ Division of Digestive Health and Liver Diseases, University of Miami Miller School of Medicine, Miami, FL, United States; ^15^ Escola Bahiana de Medicina e Saúde Pública, Salvador, Brazil

**Keywords:** bezafibrate, ciprofibrate, fibrate, primary biliary cholangitis, treatment failure, ursodeoxycholic acid

## Abstract

**Aim:** Up to 40% of patients with primary biliary cholangitis (PBC) will have a suboptimal biochemical response to ursodeoxycholic acid (UDCA), which can be improved by the addition of fibrates. This exploratory study aims to evaluate the long-term real-life biochemical response of different fibrates, including ciprofibrate, in subjects with UDCA-unresponsive PBC.

**Methods:** The Brazilian Cholestasis Study Group multicenter database was reviewed to assess the response rates to UDCA plus fibrates in patients with UDCA-unresponsive PBC 1 and 2 years after treatment initiation by different validated criteria.

**Results:** In total, 27 patients (100% women, mean age 48.9 ± 9.2 years) with PBC were included. Overall response rates to fibrates by each validated criterion varied from 39 to 60% and 39–76% at 12 and 24 months after treatment combination, respectively. Combination therapy resulted in a significant decrease in ALT and ALP only after 2 years, while GGT significantly improved in the first year of treatment. Treatment response rates at 1 and 2 years appear to be comparable between ciprofibrate and bezafibrate using all available criteria.

**Conclusion:** Our findings endorse the efficacy of fibrate add-on treatment in PBC patients with suboptimal response to UDCA. Ciprofibrate appears to be at least as effective as bezafibrate and should be assessed in large clinical trials as a possibly new, cheaper, and promising option for treatment of UDCA-unresponsive PBC patients.

## Introduction

Primary biliary cholangitis (PBC) is a cholestatic liver disorder of unknown cause that may progress to cirrhosis and liver failure (Lleo et al., 2020). Treatment with ursodeoxycholic acid (UDCA) has been shown to improve transplantation-free survival, particularly in subjects with biochemical response assessed 1 year after treatment (Harms et al., 2019; Montano–Loza and Corpechot, 2020). However, more than one-third of the patients with PBC do not respond to UDCA (Montano–Loza and Corpechot, 2020). Recently, add-on therapy with fibrates was shown to improve treatment responses to UDCA in refractory patients (Ghonem and Boyer, 2013; Grigorian et al., 2015; Corpechot et al., 2018; Reig et al., 2018). Fibrates are peroxisome proliferator–activated receptor (PPAR) agonists and are FDA approved for treatment of dyslipidemia. PPARs are a family of ligand-dependent transcription factors composed of three subtypes PPARα, PPARβ/δ, and PPARγ with different functions, distributions, affinities, and specificities for their ligands. Each of them has distinct pleiotropic roles in the modulation of energy, lipid, cholesterol, and bile acid homeostasis (Ghonem et al., 2015; Tanaka et al., 2017; Monroy–Ramirez et al., 2021). In this regard, it has been demonstrated that PPARα activation is capable of modulating bile acid metabolism due to activation of genes involved in bile acid synthesis and transportation. Fenofibrate and pemafibrate, PPARα ligands, and bezafibrate, a pan-PPAR agonist, were shown to improve treatment response to UDCA in several uncontrolled randomized controlled trials (RCTs) (Ghonem and Boyer, 2013; Grigorian et al., 2015; Reig et al., 2018) and at least one RCT (Corpechot et al., 2018). Pruritus was also significantly improved in subjects with PBC (Reig et al., 2018) and primary sclerosing cholangitis (de Vries et al., 2021) treated with bezafibrate. Most of the studies evaluating the use of fibrates in cholestatic liver diseases employed either one of those drugs; the use of ciprofibrate, another PPARα agonist, has not yet been evaluated in patients with PBC. The purpose of this exploratory study was to evaluate the long-term real-life biochemical response of different fibrates in subjects with PBC unresponsive to UDCA.

## Methods

### Study Population

The Brazilian Cholestasis Study Group (BCSG) is a multicenter collaborative consortium of investigators from academic institutions and community-based sites that treat patients with PBC in Brazil. The study population included adult (aged ≥18 years) patients diagnosed with PBC between January 1st 1992 and December 31st^,^ 2019 in 28 hepatology centers across the country. All study procedures were conducted in accordance with the ethical standards of the Helsinki Declaration. The present study was approved by the Federal University of Minas Gerais Ethics Committee Board (CAAE 98627218.6.1001.5149), and individual informed consent was waived as this study was retrospective in design. Diagnosis of PBC was considered if patients fulfilled at least two of the following three diagnostic criteria for PBC as recommended by the American Association for the Study of Liver Diseases guidelines: 1) positive serology for anti-mitochondrial antibodies (AMA) or PBC-specific antinuclear antibodies (ANA); 2) persistent increase in the serum alkaline phosphatase (ALP) level; and 3) liver histology compatible with PBC (Lindor et al., 2019). Patients in whom the diagnosis could not be confirmed or who had another etiology of liver disease were excluded.

### Data Collection

Each investigator was asked to identify all PBC patients followed up in their center at the time of the survey, without any selection or exclusion whatsoever, and to fill in a standardized database provided by the BCSG. Patients unresponsive to UDCA after at least 1 year of treatment were identified in the database and those individuals treated with fibrates enrolled in this study. Data on liver enzymes, including alanine aminotransferase (ALT), aspartate aminotransferase (AST), gamma-glutamyl transferase (GGT), and ALP, were collected at baseline and 12 and 24 months after fibrate add-on therapy for paired analysis. Biochemical results were normalized by upper limit of normal (ULN) to homogenize data interpretation. The considered standardized daily dose of UDCA for PBC treatment was 13–15 mg/kg of body weight. Lack of response to UDCA treatment was analyzed according to local investigator discretion using either one of the following criteria: Barcelona, Paris 1 and 2, Toronto, Rotterdam, and POISE trial at different time points. The duration of follow-up was defined as the interval between the diagnosis and the last visit or the date of liver transplantation or death. Advanced PBC was defined by the presence of moderate to severe fibrosis (Ludwig stage III or IV) on liver histology (when available) or clinical evidence of cirrhosis. All patients with cirrhosis were Child–Pugh A and had compensated disease.

### Statistical Analysis

Statistical analysis was performed using SPSS 25.0 software (IBM, United States). Continuous variables distribution was assessed by the Shapiro–Wilk test, and those with Gaussian distribution were expressed as mean and standard deviation, or as median and interquartile range (IQR) in case of skewed distribution. Categorical variables were expressed as absolute number and percentage. Univariate analysis was performed using chi-square, Fisher’s exact, or McNemar’s test, as appropriate, for categorical variables. Continuous variables were analyzed by the Student *t*-test or Mann–Whitney *U*-test, according to the distribution. A *p*-value < 0.05 was considered significant.

## Results

### Patient Characteristics

The clinical and laboratory features and treatment outcomes of the entire cohort of 482 Brazilian patients with PBC were previously described (Cançado et al., 2022). Fifty-nine patients with inadequate response to UDCA received add-on therapy with fibrates. Twenty-seven of the 59 patients had paired results of liver enzymes at baseline and 1 and 2 years after treatment with bezafibrate (*n* = 9) or ciprofibrate (*n* = 18) and were included in this analysis (Table 1). Briefly, all patients were women, with a mean age at diagnosis of 48.9 ± 9.2 years. Based on histological or clinical and laboratory findings, 29.6% of them had advanced PBC disease. The mean time of UDCA treatment before add-on therapy with fibrates was 19.7 ± 10.6 months. All patients were followed up for a mean period of 67 ± 35 months. The mean dose of bezafibrate was 358.3 ± 82.1 mg/day, while that of ciprofibrate was 100 mg/day. Two (7.4%) patients died and 1 (3.7%) required liver transplantation during the follow-up.

**TABLE 1:** Baseline characteristics of patients with primary biliary cholangitis using fibrates.

Variable	*N = 27*
Age at diagnosis (years ± SD)	48.9 ± 9.2
Female	100%
Autoantibody	
AMA-positive	88.9%
ANA-positive	85.2%
Symptoms at diagnosis	
Asymptomatic	33.3%
Pruritus	44.4%
Fatigue	44.4%
Coexistent autoimmune diseases	
Hashimoto thyroiditis	14.8%
Sjogren syndrome	11.1%
CREST syndrome	3.7%
Histological disease stage, n (%)	20 (74.1)
Stage I	25%
Stage II	40%
Stage III	25%
Stage IV	10%
Follow-up time (months ± SD)	67 ± 35
Advanced PBC	29.6%
Liver transplantation during follow-up	3.7%
Death	7.4%

### Response to Fibrates

**FIGURE 1:**
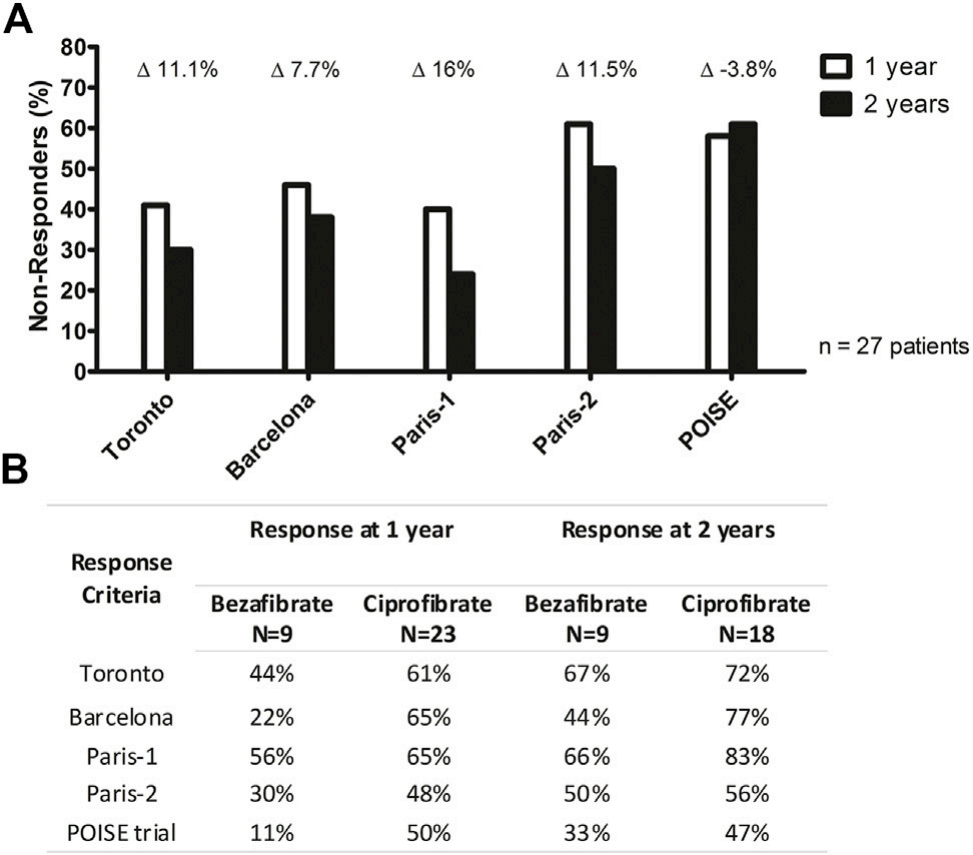
**(A)** Paired biochemical global response rate stratified by different criteria 1 and 2 years after associating fibrates to UDCA. Deltas show the percentage response gain between 1 and 2 years of treatment. **(B)** Comparison between bezafibrate and ciprofibrate response to treatment according to different criteria 1 and 2 years after associating fibrates to UDCA.

Overall response rates to fibrates by each validated criterion at 12 and 24 months are shown in Figure 1A. The proportion of nonresponders to treatment continued to reduce after 1 year of treatment with fibrates, reaching lower values at 24 months. ALP levels diminished at any degree in 59.4% of the patients after 12 months and in 66.7% after 24 months. Combination therapy resulted in a statistically significant decrease in AST/ULN and ALP/ULN only after 2 years, while GGT/ULN significantly improved in the first year of treatment (Table 2). Treatment response rates at 1 and 2 years for the ciprofibrate and bezafibrate groups are shown in Figure 1B.

**TABLE 2:** Median paired biochemical changes overtime after the introduction of fibrates

Time of measurement	AST/ULN (*n* = 27)	AST/ULN percentage difference from baseline	*p*-value	*p*-value
			Comparison with baseline values	Comparison with last measurement
Baseline	1.42	—	—	
1 year	1.10	−25.4%	0.353	0.353
2 years	1.06	−26.9%	0.052	0.010
Time of measurement	ALT/ULN (*n* = 27)	ALT/ULN percentage difference from baseline	*p*-value	*p*-value
			Comparison with baseline values	Comparison with last measurement
Baseline	1.52	—	—	
1 year	1.13	−25.7%	0.287	0.287
2 years	1.06	−30%	0.030	0.101
Time of measurement	ALP/ULN (*n* = 27)	ALP/ULN percentage difference from baseline	*p*-value	*p*-value
			Comparison with baseline values	Comparison with last measurement
Baseline	1.73	—	—	
1 year	1.61	−7%	0.304	0.304
2 years	1.36	−21.4%	0.021	0.225
Time of measurement	GGT/ULN (*n* = 27)	GGT/ULN percentage difference from baseline	*p*-value	*p*-value
			Comparison with baseline values	Comparison with last measurement
Baseline	4.60	—	—	
1 year	3.22	−30%	0.048	0.048
2 years	3.08	−33%	0.036	0.278
Time of measurement	TB/ULN (*n* = 27)	TB/ULN percentage difference from baseline	*p*-value	*p*-value
			Comparison with baseline values	Comparison with last measurement
Baseline	0.54	—	—	
1 year	0.55	+1.8%	0.647	0.647
2 years	0.43	−20.4%	0.820	0.386

AST, aspartate aminotransferase; ULN, upper limit of normal; UDCA, ursodeoxycholic acid; ALT, alanine aminotransferase; ALP, alkaline phosphatase; GGT, gamma-glutamyl transferase; TB, total bilirubin. Data are expressed as ratio between serum measurement and ULN. Wilcoxon test was performed.

No differences in response rates by different criteria at 12 and 24 months of therapy were observed when comparing patients with AMA-positive *vs* AMA-negative PBC for response to treatment, except for the Barcelona criteria at 24 months, in which AMA-positive patients were more likely to achieve response to treatment (71.4% *vs* 28.6%, *p* = 0.042). Biochemical changes stratified by the presence and absence of advanced PBC are presented in Supplementary Table S1.

## Discussion

About 40% of the patients will not have an optimal response to UDCA and are at a higher risk for disease progression to cirrhosis and liver failure. In this study, we have shown that more than half of those patients with PBC previously unresponsive to UDCA using different criteria had 1-year biochemical response with add-on therapy with either ciprofibrate or bezafibrate. Most of the patients with PBC were treated with ciprofibrate because this drug is currently offered free of charge by Brazil’s unified health system (Sistema Único de Saúde, SUS) to treat dyslipidemia. To our knowledge, this is the first report on the use of ciprofibrate in subjects with PBC, suggesting that treatment response to those drugs is not restricted to bezafibrate or fenofibrate (Ghonem and Boyer, 2013; Grigorian et al., 2015; Corpechot et al., 2018; Reig et al., 2018) and may in fact be due to a class effect. This is in accordance with a recent pilot study evaluating the use of another fibrate in patients with PBC, which reported more than 50% reduction in ALP associated with the use of pemafibrate (Joshita et al., 2019).

Recently, the combination of UDCA with bezafibrate was associated with a lower risk of all-cause and liver-related mortality or need for liver transplantation (Tanaka et al., 2021). In contrast to the BEZURSO trial (Corpechot et al., 2018), which showed up to 60% reduction in ALP after only 3 months of add-on bezafibrate therapy, biochemical response in the present study was much slower, with only 21.4% reduction in ALP after 2 years of add-on fibrate therapy. Although other studies (Kurihara et al., 2000; Nakai et al., 2000; Itakura et al., 2004) also reported a greater reduction in ALP over time with the use of UDCA associated with fibrates, some observed a much lower reduction (Liberopoulos et al., 2010; Cheung et al., 2016). This may be explained by different baseline alkaline phosphatase levels and by the proportion of patients with advanced PBC included in the aforementioned studies, which may impact the frequency and timing of biochemical response to treatment.

Each fibrate differs in its specificity for the different PPAR subtypes, α, β/δ, and γ. The mechanism(s) by which fibrates reduce biochemical markers of cholestasis remains unclear, but experimental studies have shown that they may have different roles in the regulation of bile acid (BA) synthesis and secretion. Ciprofibrate, a PPARα agonist, has been previously shown to downregulate the mRNA expression of BA-synthesizing enzymes—cytochrome P450 (CYP) cholesterol 7A1-hydroxylase (CYP7A1) and cytochrome sterol 27-hydroxylase (CYP27A1). Furthermore, it induces the promoter activity of the human apical sodium-dependent bile salt transporter (ASBT) gene in Caco-2 cells and upregulates hepatic mRNA Mdr1a/b in wild-type mice. On the other hand, bezafibrate, a dual PPAR and pregnane receptor X agonist, increases the mRNA expression of sodium taurocholate cotransporting polypeptide (NTCP), CYP3A4, multidrug resistance proteins 1 and 3, and multidrug resistance–associated protein 2 (MRP2), while downregulating the expression of CYP7A1 and CYP27A1 in human hepatoma cells [reviewed in Ghonem et al. (2015)].

Our study has limitations, including its retrospective design, lack of data regarding adverse effects, and limited number of patients. Although safety and tolerability have been previously described for bezafibrate and fenofibrate in patients with PBC and primary sclerosing cholangitis (Carrion et al., 2021), safety data regarding ciprofibrate use in humans have only been described in patients with dyslipidemia (Betteridge and O'Bryan-Tear, 1996).

In summary, our findings support the efficacy of fibrate add-on treatment in PBC patients with suboptimal response to UDCA. Although we cannot conclude on the effectiveness of ciprofibrate for UDCA-unresponsive PBC, nor confirm its safety, this investigation provides a proof of concept of a new and possibly cheaper alternative for treating these patients, since ciprofibrate appears to be at least as effective as bezafibrate. Ciprofibrate should be assessed in large prospective clinical trials as a promising option for the treatment of UDCA-unresponsive PBC patients.

## Data Availability

The original contributions presented in the study are included in the article/Supplementary Material. Further inquiries can be directed to the corresponding author.

## Abbreviations

ALP, alkaline phosphatase; ALT, alanine aminotransferase; AMA, anti-mitochondrial antibodies; ANA, antinuclear antibodies; AST, aspartate aminotransferase; BCSG, Brazilian Cholestasis Study Group; GGT, gamma-glutamyl transferase; IQR, interquartile range; PBC, primary biliary cholangitis; PPAR, peroxisome proliferator–activated receptor; SD, standard deviation; UDCA, ursodeoxycholic acid.
