# Supplementary material for: Fibrates for the Treatment of Primary Biliary Cholangitis Unresponsive to Ursodeoxycholic Acid: An Exploratory Study
Source: Front Pharmacol. 2022 Jan 20;12:818089. doi: 10.3389/fphar.2021.818089 (PMC8811361; doi:10.3389/fphar.2021.818089)
Supplement: Supplementary file 1 [file Table1.docx]

**Supplementary Table 1: Biochemical changes overtime during fibrate treatment stratified by the presence of advanced PBC.**

|  | **Initial disease (n = 19)** | | | **Advanced disease (n = 8)** | | |
| --- | --- | --- | --- | --- | --- | --- |
|  | **Baseline** | **12 mo.** | **24 mo.** | **Baseline** | **12 mo.** | **24 mo.** |
| **AST/ULN** | 1.43 | 1.10 | 1.13 | 1.12 | 1.13 | 0.83 |
| **ALT/ULN** | 1.74 | 1.03 | 1.11 | 0.97 | 0.9 | 0.77 |
| **ALP/ULN** | 1.60 | 1.19 | 1.10 | 2.0 | 1.82 | 1.07 |
| **GGT/ULN** | 3.85 | 2.42 | 2.66 | 4.22 | 3.02 | 3.08 |
| **TB/ULN** | 0.54 | 0.51 | 0.51 | 0.52 | 0.73 | 0.43 |

AST, aspartate aminotransferase; ALT, alanine aminotransferase; ALP, alkaline phosphatase; GGT, gammaglutamyl-transferase; Mo., months; TB, total bilirubin; ULN, upper limit of normal. Data are expressed as median serum levels divided to the upper limit of normal.
